# Swellable Copolymers of *N*-isopropylacrylamide and Alkyl Acrylic Acids for Optical pH Sensing

**DOI:** 10.3390/molecules25061408

**Published:** 2020-03-19

**Authors:** Barry K. Lavine, Sandhya R. Pampati, Kaushalya S. Dahal, Mariya Kim, U. D. Nuwan T. Perera, Marcus Benjamin, Richard A. Bunce

**Affiliations:** 1 Department of Chemistry, Oklahoma State University, Stillwater, OK 74078, USA; sandhya.pampati@okstate.edu (S.R.P.); kaushas@okstate.edu (K.S.D.); mashakim2003@yahoo.com (M.K.); marcus.benjamin@okstate.edu (M.B.); richard.a.bunce@okstate.edu (R.A.B.); 2 Department of Chemistry and Physics, 231 Natural Sciences Building, 111 Memorial Drive, Western Carolina University, Cullowhee, NC 28723, USA; uperera@email.wcu.edu

**Keywords:** polymer swelling, *N*-isopropylacrylamide hydrogels, optical pH sensing, turbidimetry, enthalpy and entropy of pH induced swelling

## Abstract

Swellable polymers that respond to pH (including a portion of the physiological pH range) have been prepared from *N*-isopropylacrylamide (NIPA) copolymerized with acrylic acid, methacrylic acid, ethacrylic acid or propacrylic acid by dispersion polymerization. When the swellable polymer particles are dispersed in a polyvinyl alcohol (PVA) hydrogel membrane, large changes occur in the turbidity of the membrane (which is measured using an absorbance spectrometer) as the pH of the buffer solution in contact with the hydrogel membrane is varied. The swelling of the NIPA copolymer is nonionic, as the ionic strength of the buffer solution in contact with the PVA membrane was increased from 0.1 to 1.0 M without a decrease in the swelling. For many of these NIPA copolymers, swelling was also reversible in both low- and high ionic strength pH-buffered media and at ambient and physiological temperatures. The composition of the formulation used to prepare these copolymers of NIPA can be correlated to the enthalpy and entropy of the pH-induced swelling.

## 1. Introduction

pH is routinely measured in the laboratory using a glass electrode [1]. The necessity for continued recalibration and the dependence of the liquid junction potential on solution composition and concentration have limited the application of glass electrodes in clinical, biomedical, and biotechnological analyses. Although there has been considerable interest in the in-vivo pH sensing of blood during surgery for patients who suffer from tissue ischemia [2], the glass electrode is too bulky for invasive tissue analysis. Glass pH electrodes are also not suitable to the study of gastroesophageal reflux disease [3] because of a lack of stability of the calibration over an extended period of time. As pH is a ubiquitous indicator of cell growth and metabolism, monitoring and controlling pH in fermentation baths [4,5,6] is crucial. Although conventional glass pH electrodes have been adapted to fermentation reactions and bioreactors, they suffer from several drawbacks, including the necessity for pressurized compensation of the electrolyte and interference by other components (e.g., proteinaceous materials) in the medium.

For these reasons, optical pH sensing implemented through fiber optics has attracted considerable attention. The term optrode is often used to describe these sensors. pH optrodes usually consist of a pH indicator bound onto a solid support or immobilized in a polymer matrix at the distal end of an optical fiber [7]. Interaction of the indicator at the end of the fiber with the sample leads to changes in the optical properties of the indicator which is detected through the optical fiber by absorbance or fluorescence from the sensing material [8,9]. Optrodes have several advantages over glass electrodes including low cost, small size and the robustness of the optical fiber. However, the use of chromophores (e.g., pH sensitive dyes as indicators) also has its drawbacks. The reagent may leach out over time and chromophores can photodegrade, limiting the lifetime of the sensor. The chromophores are wavelength-specific, imposing restrictions on the instrumentation that can be used. Furthermore, optrodes based on the fluorescence of chromophores typically generate smaller responses.

To overcome these disadvantages, optrodes utilizing swellable polymers functionalized to respond to pH have been developed [10,11,12,13,14,15,16]. Polymer swelling has several advantages over detection methods involving chromophores. The analyte signal is based on turbidity and is wavelength-independent. The problem of photodegradation is obviated, and near infrared (NIR) wavelengths, which can be used to monitor changes in the turbidity of the swellable polymer, can be transmitted through an optical fiber without appreciable attenuation, unlike the ultraviolet or visible electromagnetic radiation used in absorption or fluorescence.

Several studies [13,14,15,16] which have been undertaken on polymer swelling for optrodes have focused on the development of formulations to prepare derivatized polystyrene that can undergo a large number of swelling and shrinking cycles without cracking. The optical properties of the derivatized polystyrene changes with swelling in aqueous solution. In the unswollen state, the polymer is turbid, whereas the polymer is more transparent in the swollen state. The reason for this is that derivatized polystyrene contains large pores filled with water. Since water has a lower refractive index than the polymer, these pores will both reflect and scatter light. When the polystyrene particles swell, the amount of water in the pores of the polymer increases and the refractive index decreases. As the refractive index of the polymer particles approaches that of water, the polymer both reflects and scatters less light. As this is the reason for the change in the optical properties of the polymer that accompanies swelling, a better approach would involve reversing the phases, i.e., preparing microparticles and embedding them in a hydrogel membrane [17] instead of synthesizing a polymer with water-filled pores.

There are several advantages in preparing microparticles embedded in a hydrogel membrane. The weight fraction of the microparticles in the membrane is only on the order of a few percent. This means that less analyte has to react with the membrane to cause an optical change. The polymer is also free to swell in all three directions (x, y, and z) when the polymer particles are embedded in a hydrogel membrane. For chemical sensing, derivatized polystyrene particles with water-filled pores have been attached to a glass substrate in an optical reflectance device [15] or at the distal end of an optical fiber [13,14]. This prevents them from swelling parallel to the surface as they can only swell perpendicular to the substrate. Since the volume change is equal to the cube of the swelling ratio in one dimension when the polymer is free to swell in all directions, there is a large increase in sensitivity when the microparticles are embedded in a hydrogel membrane. Furthermore, the microparticles are protected from direct contact with the sample by the hydrogel membrane, which also serves as a “filter” to reject proteinaceous materials or other large macromolecular compounds present in the medium. The polymer particles will not leach out of the membrane, and the membranes have been shown to be stable for several years [17].

Previously published studies on swellable polymers for chemical sensing have primarily focused on charged polymers. The swelling of a charged polymer can be viewed as an osmotic pressure effect that results from differences between the charge density of the bulk polymer and the surrounding solution [18]. Low ionic strength solutions promote ionic swelling because of an absence of shielding of the charges by the solvent ions. In these solutions, large differences exist between the charge density of the bulk polymer and the surrounding solution. In high ionic strength solutions, however, shielding of the charges on the polymer backbone occurs. In these solutions, the differences between the charge density of the bulk polymer and the surrounding solution are smaller, which causes a reduction in swelling.

By comparison, the swelling of pH sensitive *N*-isopropylacrylamide (NIPA) polymers (which are investigated in this study), is not suppressed by the ionic strength of the solution in contact with the polymer, as swelling is both nonionic and controlled by the polymer solvent interaction parameter [19,20]. This is a distinct advantage of *N*-isopropylacrylamide (NIPA) polymers over other pH-sensitive swellable polymers previously investigated for sensor applications whose use is restricted to low ionic strength media. Another advantage is that copolymers of NIPA sensitized to pH using the appropriate functional comonomer undergo significant swelling and shrinking as a function of the pH of the solution in contact with the polymer. Furthermore, swelling is accompanied by a change in the refractive index of the polyNIPA particles from a transparent state (where the refractive index of the particles and the solvent are equal) to a milky state, due to a large amount of light scattered when the polymer is in its shrunken state.

In this study, swellable polymers that respond to pH have been prepared from NIPA copolymerized with acrylic acid (AA), methacrylic acid (MAA), ethacrylic acid (EAA), or propacrylic acid (PAA) to develop materials that respond over a large pH range, including a portion of the physiological pH range. When these NIPA polymer particles are embedded in a polyvinyl alcohol (PVA) hydrogel membrane, large changes in the turbidity of the membrane (measured by an absorbance spectrometer) occur as the pH of the buffer solution in contact with the hydrogel membrane is varied. Changes of approximately 0.3 absorbance units are observed in the swelling and shrinking of the pH-sensitive NIPA polymer particles dispersed in the membrane. Swelling of the NIPA polymer particles is nonionic as the ionic strength of the buffer solution in contact with the PVA membrane was increased from 0.1 to 1.0 M without a decrease in swelling. For many of these NIPA copolymer particles, swelling is also reversible in both low- and high ionic strength pH buffered media and at ambient and physiological temperatures.

It is the goal of this study to gain an understanding of how changes in the composition of the formulation influence the response of the functionalized polyNIPA particles to changes in the pH of the buffered media in contact with them. In particular, we seek to better understand the relationship between monomer content and type, and how changes in the composition of the formulation impact the pH response and allows the response to be tuned. The incorporation of a pH-sensitive functional co-monomer in the polymer formulation imparts unique properties to polyNIPA. For this reason, the characterization of several NIPA copolymers of acrylic acid (AA), methacrylic acid (MAA), ethacrylic acid (EAA), and propacrylic acid (PAA) was undertaken in this study. Unlike previous studies on pH-initiated swelling of acrylamide gels, which were limited to a specific pH-sensitive comonomer, a wide range of alkyl acrylic acids were investigated. Furthermore, the temperature-activated pH response of these gels has been examined in far greater detail than in previous studies, as sufficient data were available to compute the change in both the enthalpy and entropy associated with pH-induced polymer swelling.

## 2. Results

**Figure 1:**
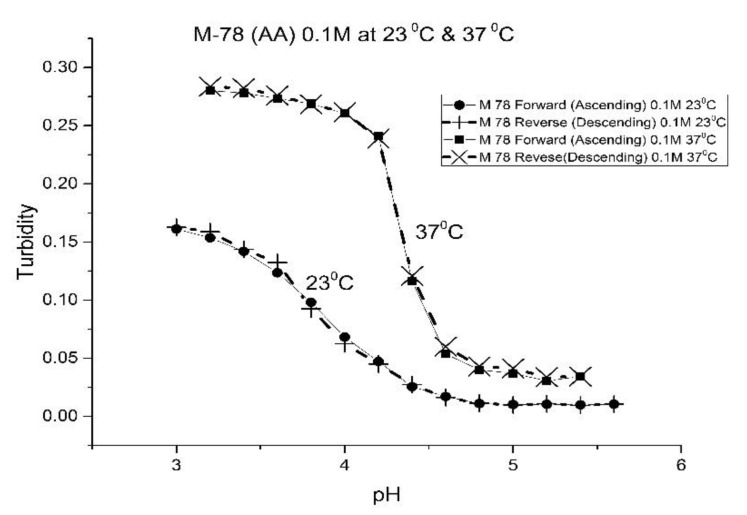
Ascending and descending pH profiles of polymer particles of M-78 (NIPA copolymerized with AA) at 23 °C and 37 °C. Solid line = ascending pH profile. Dashed line = descending pH profile.

**Figure 2:**
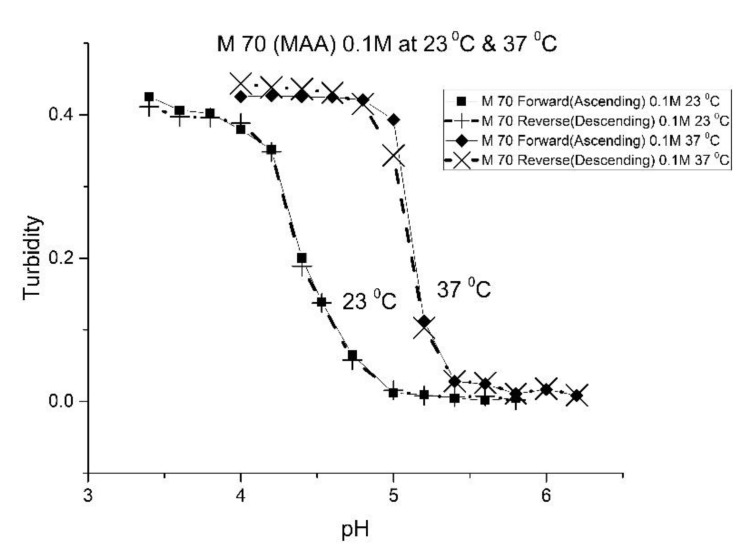
Ascending and descending pH profiles of polymer particles of M-70 (NIPA copolymerized with MAA) at 23 °C and 37 °C. Solid line = ascending pH profile. Dashed line = descending pH profile.

**Figure 3:**
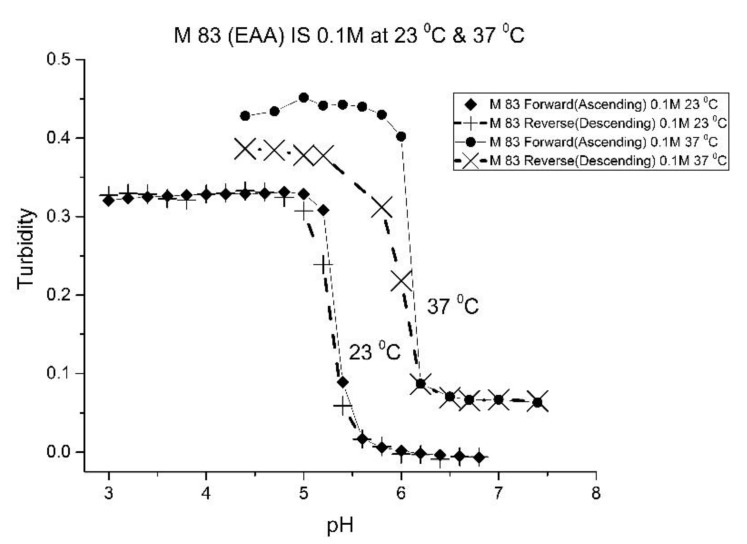
Ascending and descending pH profiles of polymer particles of M-83 (NIPA copolymerized with EAA) at 23 °C and 37 °C. Solid line = ascending pH profile. Dashed line = descending pH profile.

**Figure 4:**
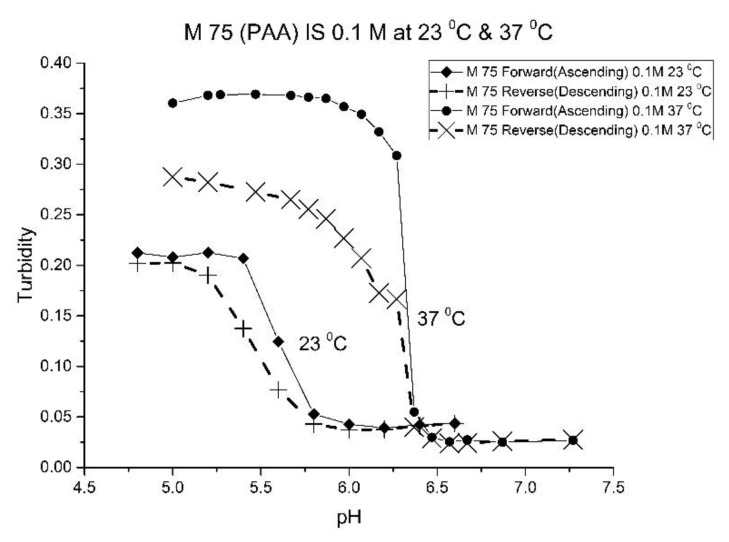
Ascending and descending pH profiles of polymer particles of M-75 (NIPA copolymerized with PAA) at 23 °C and 37 °C. Solid line = ascending pH profile. Dashed line = descending pH profile.

Figure 1, Figure 2, Figure 3 and Figure 4 show the pH response profile of four polymers prepared by the copolymerization of NIPA with AA, MAA, EAA, and PAA. The turbidity (absorbance value) collected at 700 nm is plotted against the pH of the buffer solutions in contact with the pH-sensitive hydrogel. Each polymer, whose formulation is summarized in Table 1, was embedded in a PVA hydrogel membrane. A unique aspect of this study is the use of light crosslinking in the preparation of these NIPA polymers, as many of the formulations used limited the amount of crosslinker, *N*,*N*-methylene bisacrylamide (MBA), to 5%. For each datapoint, the membrane is rinsed three times with a buffer solution of the proscribed pH and is allowed to equilibrate for 15 min prior to analysis by turbidimetry. Although the response time of the pH polymer particles is dependent on both the buffer capacity of the solution in contact with the membrane and the percentage of the alkyl acrylic acid in the copolymer, we chose to wait 15 min for each measurement to ensure that a full-scale response was always obtained. Lowering the percentage of the alkyl acrylic acid in the formulation can lead to even faster response times. For some membranes, a full-scale response to the change in pH was obtained in as little as five minutes.

For AA and MAA, swelling is reversible at 23 and 37 °C, as both the ascending (solid line) and descending (dashed line) pH profiles are superimposable, whereas swelling for the EAA copolymer is reversible only at 23 °C, and swelling for the PAA copolymer is irreversible at both temperatures. At a low pH, the polymer exists in a shrunken state and the water content of the polyNIPA particles is low. Turbidity, as measured by the UV/visible absorbance spectrometer, is high because the refractive index of the particles is higher than the poly vinyl alcohol (PVA) hydrogel membrane. The result is a loss of transmitted light due to reflection, which translates into higher absorbance values. At higher pH values, the water content of the polymer increases, due to swelling induced by the deprotonation of the pH-sensitive alkyl acrylic acid co-monomer. The refractive index of the polyNIPA particles decreases as the particles swell and approaches the refractive index of the PVA hydrogel, which is approximately 90% water. Eventually, the turbidity reaches a limiting value that corresponds to the maximum swelling of the polymer.

The inflection point in the plot of turbidity (absorbance) versus pH (see Figure 1, Figure 2, Figure 3 and Figure 4) is the apparent pK_a_ of the polymer. The term “apparent pK_a_” is used to refer to the point where the response is halfway between the response at a low pH and at a high pH, as turbidity versus pH curves have not yet been described by theory in a manner that would allow for calculation of the pK_a_ from the observed data. The change in the pH response of the membrane occurs over a narrower range than a typical pH indicator (which is plus or minus one pH unit). In all likelihood, only partial deprotonation of the carboxylic acid is necessary to generate maximum polymer swelling. Lowering the percentage of the alkyl acrylic acid in the NIPA formulation may yield polymer particles that respond over a wider pH range with a shift in their “apparent pK_a_”. The pH response range of the NIPA copolymer particles has been investigated as a function of the amount of the alkyl acrylic acid present in the formulation and is the subject of a future publication [21].

Table 2 lists the “apparent pK_a_” values of the four NIPA copolymers at 23 and 37 °C. The “apparent pK_a_”, which was computed for each pH response curve using the first derivative, can be tuned by increasing the alkyl chain length (i.e., hydrophobicity) of the pH-sensitive functional co-monomer. For the four copolymers listed in Table 1, NIPA is the dominant monomer, as there is only a small amount of pH-sensitive functional co-monomer present. For AA and MAA, NIPA is polymerized into chains containing well-separated pH-sensitive functional co-monomer units, as the relative reactivity ratios of all the monomers comprising these copolymers are approximately equal. This is probably the reason why the “apparent pK_a_” of M-78 and M-70 is similar to the pK_a_ of the AA or MAA monomers (see Table 2). Because of the lower reactivity ratio of EAA and PAA [22], the pH-functional co-monomer units in M-83 and M-75 are not as well separated. The net result is higher “apparent pK_a_” values, analogous to the higher pK_a_ values obtained for the second proton of maleic acid versus that of fumaric acid. (The “apparent pK_a_” value of M-83 and M-75 is substantially larger than the pK_a_ of the EAA or PAA monomer (see Table 2.) The greater hydrophobicity of EAA and PAA compared to AA and MAA may also be a factor, as it can result in a decrease in the water content of the polymer, resulting in higher “apparent pK_a_” values. The presence of PAA in polyNIPA also causes a decrease in the lower critical solution temperature of polyNIPA to 23 °C compared to 36.7 °C for MAA. This is attributed to the hydrophobic nature of the protonated form of PAA [23].

The increase in pK_a_ with temperature of these four NIPA copolymers is the opposite to what would occur for a dye used as a pH indicator in an optical fiber, which would be a decrease of approximately 0.1–0.3 pH units for a 10 °C increase in temperature [24]. The shift of the pK_a_ towards higher pH values can be attributed to a decrease in the water content of the acrylamide gel at higher temperatures. Park and Hoffman [25], in a previous study, have shown that a decrease in the water content of the acrylamide gel occurs as the temperature is increased. The distance between adjacent alkyl acrylic acids units, the source of charge in the NIPA copolymer, decreases due to water molecules being driven out of the polyNIPA copolymer as the temperature is increased. This, in turn, causes the apparent pK_a_ of the NIPA copolymer to increase.

The pH response of the NIPA copolymers at higher temperatures (i.e., physiological versus ambient) appears to be enhanced. Since the water content of the NIPA copolymer decreases at higher temperatures, there is an increase in the surface area to volume ratio of the polymer network. The result is that fewer protons are required to react in order to yield a full-scale pH response. Thus, the pH response appears to be enhanced.

To better understand the relationship between the “apparent pK_a_” of the pH-sensitive polyNIPA particles and temperature, pH response curves (23–40 °C) for each of these four copolymers were obtained. The “apparent pK_a_” for each response curve was computed, with the relationship between pK_a_ and temperature modeled using the van’t Hoff relationship, see Equation (1), where *K_a_* is the apparent acid dissociation constant of the NIPA copolymer, T is the temperature of the copolymer, ΔH° is the change in enthalpy associated with pH-induced swelling of the polymer, ΔS° is the change in entropy associated with the pH-induced swelling of the polymer, and R is the gas constant.

(1) $$\ln K_{a}=\frac{-{\Delta H}^{o}}{\mathrm{RT}}+\frac{{\Delta S}^{o}}{R}$$

Table 3 lists the changes in the enthalpy and entropy that occur due to pH-induced polymer swelling. The linearity of these plots over the temperature range investigated was excellent. The uncertainties in the enthalpies and entropies of swelling determined from the least squares fitting of the data shown in Table 3 are small, which is indicative of a good fit of the data. For AA, MAA, and EAA, the increase in the hydrophobicity of the co-monomer in polyNIPA can be correlated to an increase in the enthalpy and entropy of swelling for these copolymers. However, PAA does not follow this trend. The anomalous behavior of PAA may be due to the absence of *N-tert*-butyl acrylamide (NTBA) in the formulation used to prepare this NIPA copolymer.

**Figure 5:**
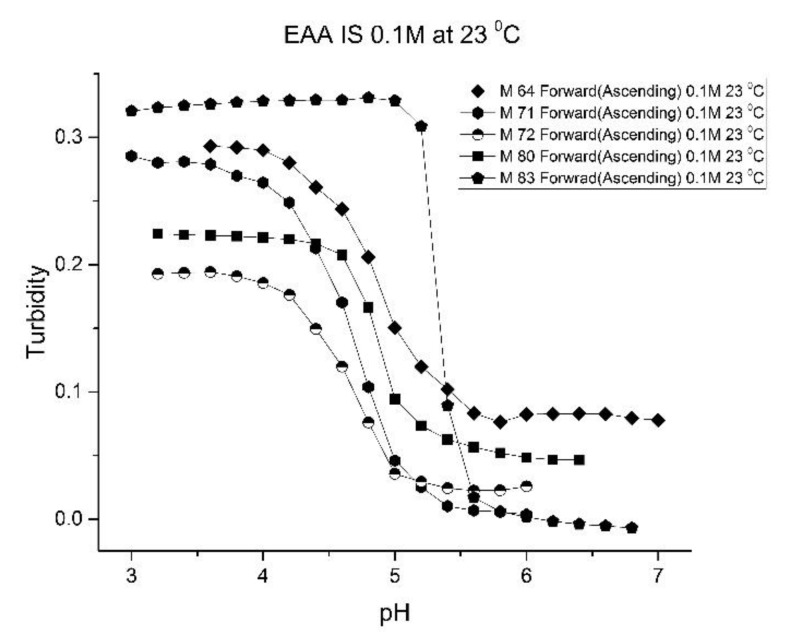
Ascending pH profiles at 23 °C for a variety of *N*-isopropylacrylamide copolymers of ethacrylic acid: M-64, M-71, M-72, M-80, and M-83.

**Figure 6:**
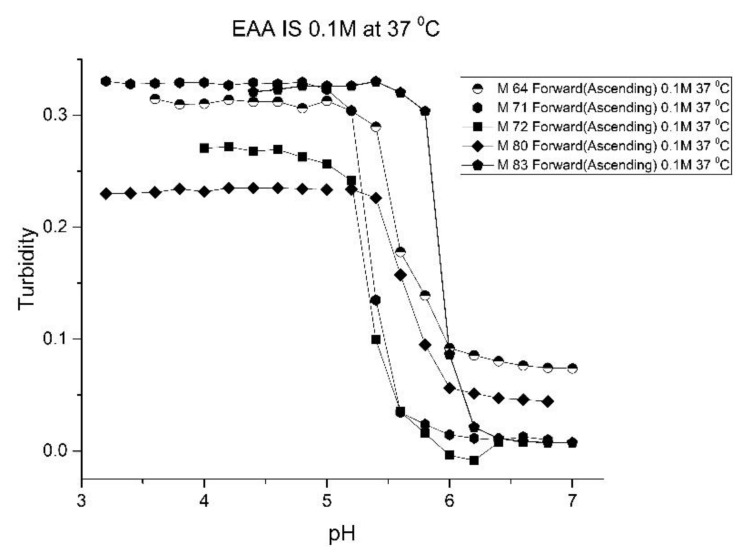
Ascending pH profiles at 37 °C for a variety of *N*-isopropylacrylamide copolymers of ethacrylic acid: M-64, M-71, M-72, M-80, and M-83.

**Figure 7:**
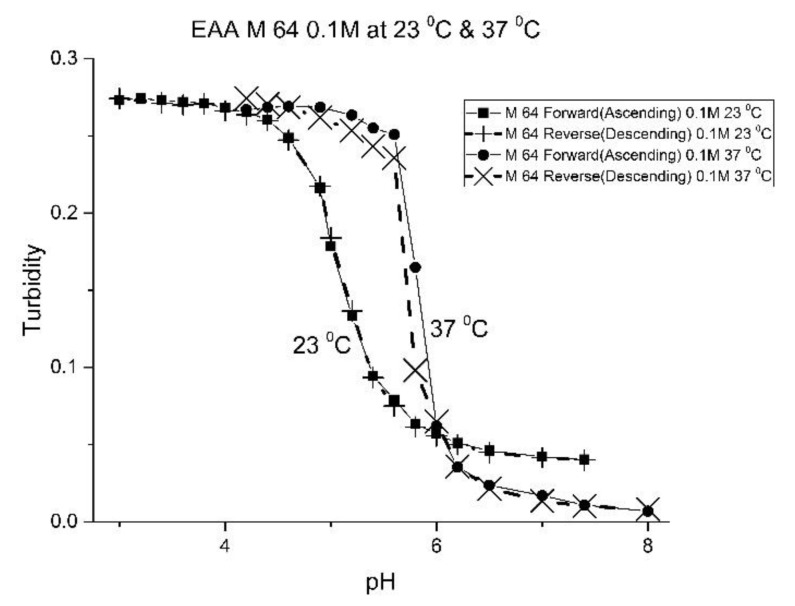
Ascending and descending pH profiles for polymer particles of M-64 at 23 °C and 37 °C. Solid line = ascending pH profile. Dashed line = descending pH profile.

As one of the motivations for undertaking this study is physiological pH sensing (pH 5–pH 7.4), we chose to focus our investigation on EAA and PAA co-polymers of NIPA. Figure 5; Figure 6 show pH response curves at 23 and 37 °C for several EAA formulations (see Table 4). From these response curves, the range of “apparent pK_a_” values spanned by the EAA co-polymers is 4.7 to 5.3 pH units at 23 °C and 5.3 to 5.9 pH units at 37 °C. The larger pK_a_ value of M-83 are attributed to the presence of NTBA in the formulation, which increases the hydrophobicity of the polymer. By varying the formulation used for these five EAA co-polymers (through adjusting the amount of MBA and NTBA in the formulation), the “apparent pK_a_” of these polyNIPA particles can be tuned (see Table 5) and the reversibility of their swelling enhanced (see Figure 7 versus Figure 3).

**Figure 8:**
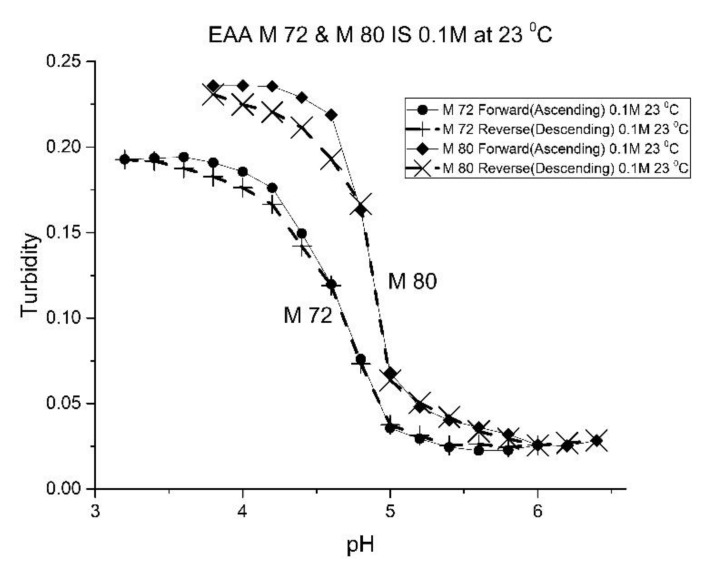
Ascending and descending pH profiles for polymer particles of M-72 and M-80 at 23 °C. Solid line = ascending pH profile. Dashed line = descending pH profile.

Although the formulation for M-72 and M-80 is the same, differences in their pH response curves (see Figure 8) and the “apparent pK_a_” values (see Table 5) obtained for these two polymers suggests that variability between batches of the same formulation is a concern in the preparation of swellable pH-sensitive polyNIPA particles. For M-72 and M-80, we attribute batch variability to the aging of some of the components used to prepare these NIPA-based copolymers. M-80 was prepared using relatively fresh components (less than one year), whereas M-72 was prepared using aged components (that were stored in a refrigerator for a few years). For acrylamide gels, the properties of the crosslinked polymers can vary between batches. However, our experience in the preparation of these NIPA copolymers is that batch variability can be mitigated by using relatively fresh components to prepare these NIPA copolymers.

The change in enthalpy and entropy of the NIPA-EAA polymer particles is summarized in Table 6. M-64, M-71, and M-72 have similar values for the entropy and enthalpy of swelling, as do M-80 and M-83. We attribute this clustering to the use of relatively fresh reagents (NIPA, NTBA, and MBA) for M-80 and M-83, whereas the formulation components used to prepare M-64, M-71, and M-72 were several years old.

**Figure 9:**
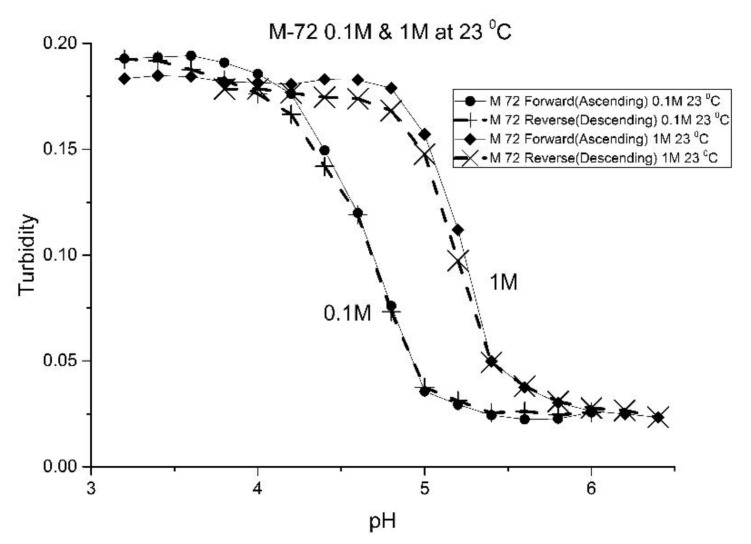
Ascending and descending pH profiles for polymer particles of M-72 at 0.1 M and 1M ionic strength and 23 °C. Solid line = ascending pH profile. Dashed line = descending pH profile.

The effect of the ionic strength of the buffer solution in contact with the EAA co-polymers was also investigated at ambient temperature. pH response curves were obtained at 0.1 M and 1.0 M ionic strength with sodium chloride used to control the ionic strength of the solution. Figure 9 shows the pH response curve at 0.1 M and 1.0 M for M-72 which is representative of the set of EAA copolymers investigated in this study.

Swelling is both significant and reversible at both 0.1 and 1.0 M ionic strength. The pH response curves at both 0.1 and 1.0 M ionic strength indicate that swelling for the EAA copolymers is nonionic. Furthermore, pH response curves of similar NIPA copolymers investigated in previous studies were unchanged by the ionic strength of the buffer solution in contact with the membrane when the ionic strength of the buffer is less than 0.5 M [17,21]. As for the increase in the apparent pK_a_ of the polyNIPA particle when the ionic strength of the buffer solution in contact with the particles is 1.0 M, the clear shift in the pH response to higher pH values can be attributed to the decrease in the water content of the acrylamide gel at high (approximately 1M NaCl) concentrations. The effect of the NaCl concentration on the water content of polyNIPA was previously reported by Park and Hoffman [25]. At high NaCl concentrations, the distance between alkyl acrylic acid monomer units (which is responsible for the source of charge in the NIPA copolymer) decreases as a result of the loss of water from the polymer. Hence, the increase in the “apparent pK_a_” of the polymer can be attributed to a decrease in the distance between pH-functional comonomer units in the three dimensional polymer network due to a decrease in the volume of the polymer, as a result of the water molecules being driven out of the NIPA copolymer at high NaCl concentrations. The fact that significant swelling is observed at high NaCl concentrations makes suitably functionalized NIPA copolymers well-suited to perform optical pH sensing in ocean and sea water.

**Figure 10:**
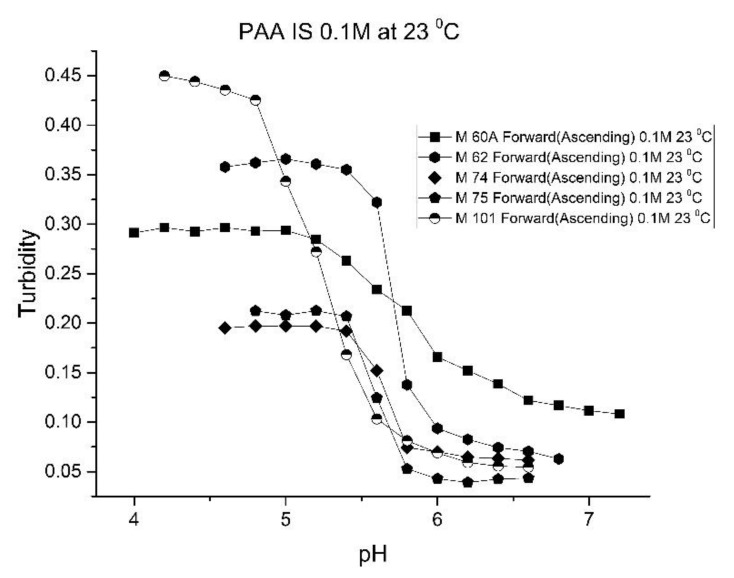
Ascending pH response curves for polymer particles of M-60A, M-62, M-74, M-75, and M-101 at 23 °C.

**Figure 11:**
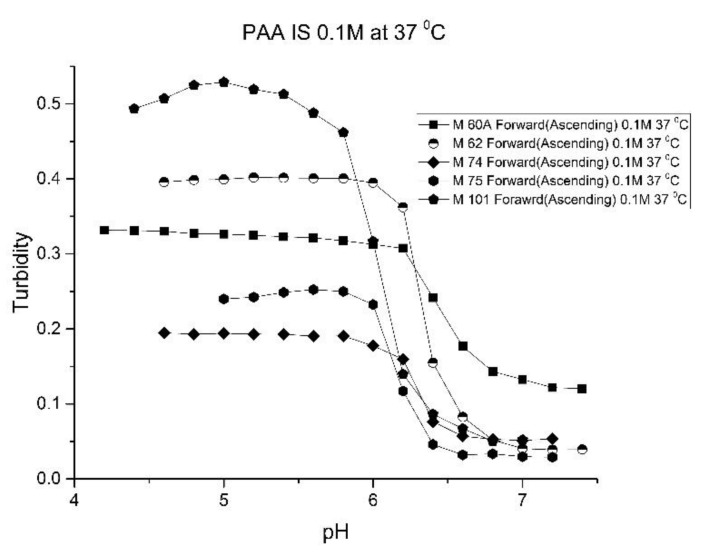
Ascending pH response curves for polymer particles of M-60A, M-62, M-74, M-75, and M-101 at 37 °C.

**Figure 12:**
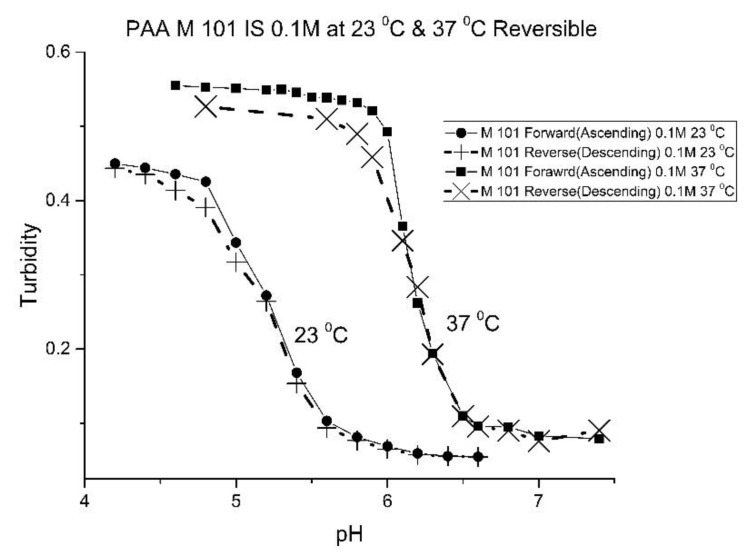
Ascending and descending pH profiles for M-101 particles at 23 and 37 °C. Solid line = ascending pH profile. Dashed line = descending pH profile.

**Figure 13:**
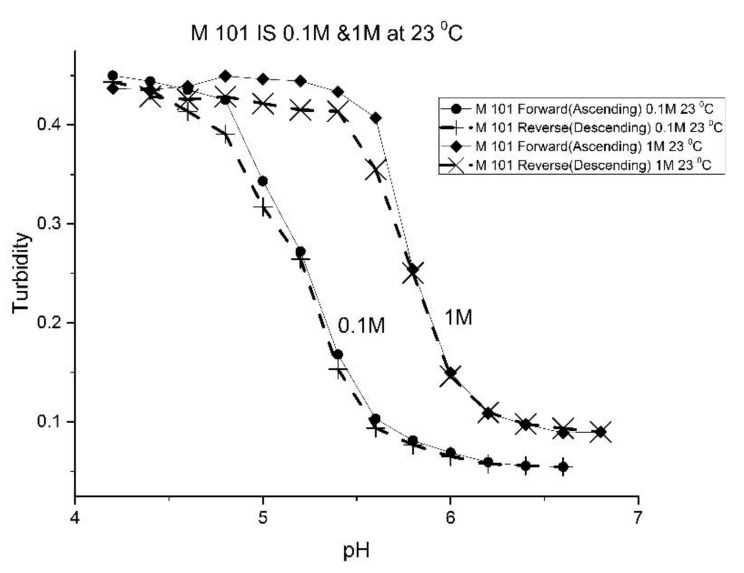
Ascending and descending pH profiles for M-101 particles at 0.1 M and 1 M ionic strength and 23 °C. Solid line = ascending pH profile. Dashed line = descending pH profile.

Figure 10, Figure 11 show ascending pH response curves at 23 and 37 °C for a variety of PAA-NIPA co-polymer formulations (see Table 7). From this set of pH response curves, the range of pK_a_ values spanned by the PAA co-polymers is 5.3 to 5.9 at 23 °C and 6.1 to 6.5 at 37 °C. By varying the formulation used to prepare these PAA co-polymers (through adjustments in the amount of the pH sensitive functional comonomer and crosslinker present in the formulation), the “apparent pK_a_” of these NIPA copolymers can be tuned (see Table 8), and the reversibility of the swelling can be enhanced (see Figure 12 versus Figure 4). The change in the enthalpy and entropy of these particles for pH-induced swelling is summarized in Table 9. M-60A, M-62, and M-74 have similar values for enthalpy and entropy, as do M-75 and M-101. The dichotomy appears to be correlated to the degree of crosslinking for this set of NIPA copolymers. The amount of crosslinker in the formulation used to prepare M-75 and M-101 is 5%, whereas the amount of crosslinker is greater than 5% in the M-60A, M-62, and M-74 polymer formulations. Figure 13 shows the effect of ionic strength on the pH response of M-101. The results obtained for M-101 are similar to those obtained for M-72 (see Figure 9).

Many of the NIPA copolymers synthesized for use in this study have undergone more than one-hundred swelling and shrinking cycles without loss of functionality. These copolymers have exhibited reproducible responses for numerous swelling and shrinking cycles (e.g., see the forward and reverse titration curves for M-64, M-70, M-72, M-78, M-80, M-83, and M-101). All regions of the corresponding titration curves, including those which show relatively low changes in turbidity for a portion of the pH range, can be reproduced for the same segment of the membrane. Swelling was also reversible for many of the NIPA copolymers investigated in this study. Therefore, any calibration will only need to be performed once for copolymers that exhibit reversible swelling. When placed in a glass vial containing distilled water and stored away in a refrigerator, these polymers have retained their full operational effectiveness, even after 5 years.

## 3. Materials and Methods

### 3.1. Materials

NIPA and acetonitrile were purchased from Acros (Morris Planes, NJ, USA). EAA and PAA were prepared using a procedure developed by Tirrell and coworkers [26]. AA, MAA, *N-tert*-butylacrylamide (NTBA), 2,2-Dimethoxy-2-phenylacetophenone (DMPA), PVA (MW 85,000–146,000, 98–99% hydrolyzed), and glutaric dialdehyde (50 w% solution in water) were purchased from Aldrich Chemical Co. (Milwaukee, WI, USA). Glutaric dialdehyde was diluted to 10% with DI water prior to use. *N*,*N*-methylenebisacrylamide (MBA) was purchased from BioRad (Hercules, CA). Acetic acid and hydrochloric acid were purchased from Pharmco, sodium chloroacetate, 2-(*N*-Morpholino)ethanesulfonic acid (MES), and 3-(*N*-Morpholino)propanesulfonic acid (MOPS) were purchased from Sigma-Aldrich (Atlanta, GA, USA), and sodium hydroxide was purchased from Thermo-Fisher (Waltham, MA, USA). Unless otherwise indicated, all reagents and solvents were used as received.

### 3.2. Synthesis of pH Sensitive PolyN-isoproplyacrylamide Particles

**Figure 14:**
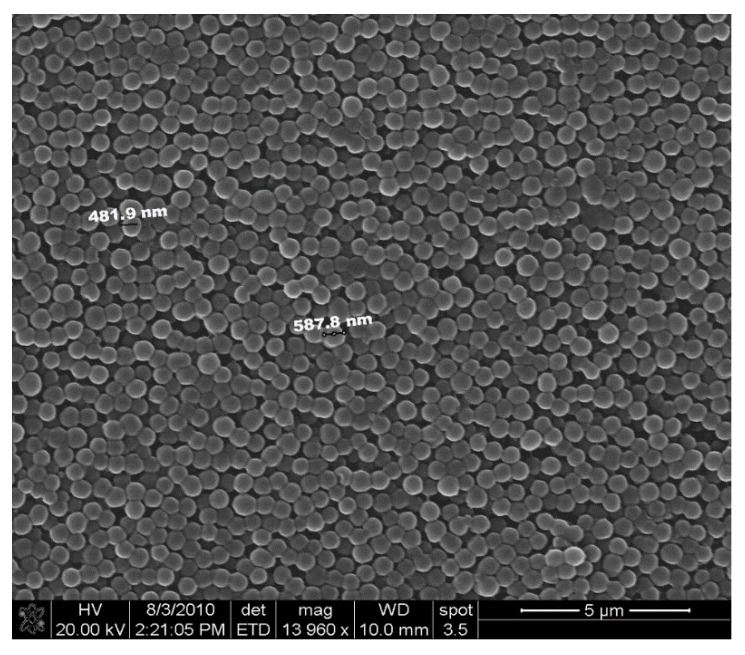
Scanning electron micrograph of polymer particles of M-70 (15 millimoles of *N*-isopropylacrylamide, 2 millimoles of methacrylic acid, 2 millimoles of *N-tert*-butyl acrylamide, and 1 millimole of *N*,*N*-methylene bisacrylamide) prepared by dispersion polymerization.

PolyNIPA particles were synthesized by free-radical-initiated dispersion photopolymerization. The components comprising the formulation used to prepare the pH-sensitive polyNIPA particles included a transduction monomer (NIPA), initiator (DMPA), functional co-monomer (AA, MA, EAA and PAA), and crosslinker (MBA). Acetonitrile was used as the solvent, as all components of the formulation are soluble in it. The formulation was prepared by adding NIPA, functional co-monomer, and MBA into a 500 mL 3-neck round-bottom Pyrex flask containing 100 mL of acetonitrile. When AA or MAA were used as the functional co-monomer, NTBA was added to the formulation. The mixture was stirred for 30 min in a closed system to prevent oxygen from infiltrating into the reaction mixture. Once the components were dissolved, 0.2 g of DMPA and the functional co-monomer were added to the reaction mixture, which was sonicated for 20 min with a Branson 1510 ultrasonicator (Branson Ultrasonics Corp, Danbury, CT, USA) while simultaneously being purged using dry nitrogen. After sonication and purging, the 500 mL 3-necked flask was placed in a Rayonet UV photolysis chamber (Southern New England Ultraviolet Company, Branford, CT) equipped with G4T5 type mercury lamps (Southern New England Ultraviolet Company, Branford, CT) and a cooling fan. The contents of the flask were stirred using a paddle to initiate polymerization. The wavelengths used were from 315–400 nm (The UV cut-off for Pyrex is below 260 nm). The reaction was carried out at ambient temperature for 12 h and. after 12 h, a turbid polymer suspension was formed. The turbid polymer suspension was transferred into two 40 mL polypropylene centrifuge tubes and centrifuged at 3000 rpm for 10 min. The decant was separated and the particles were suspended in 25 mL aliquots of acetonitrile, sonicated for 30 min and again centrifuged at 3000 rpm for 10 min. This washing procedure was repeated four times. The particle suspension was than washed with 25 mL aliquots of methanol (three times). After these washings, the particles were transferred into a vial containing methanol and stored in a refrigerator. The size of the polymer particles produced by this procedure varied from 0.5 to 1 μm. A scanning electron micrograph of a typical polymer particle prepared using this procedure is shown in Figure 14.

### 3.3. Preparation of PVA Membranes

The hydrogel membranes used to characterize the swelling of the pH-sensitive polyNIPA particles were prepared by mixing the particles with an aqueous PVA solution followed by crosslinking using glutaric dialdehyde. In a typical membrane preparation, 2 g of a mixture of the polyNIPA particles (1%, *w*/*w*) and PVA solution (8% *w*/*w*) was prepared in a 4 mL glass vial and the mixture was magnetically stirred overnight in order to homogeneously disperse the particles in the PVA solution. A total of 50 μL of 10% aqueous glutaric dialdehyde was then added using a micropipette and stirring was continued for another hour. Finally, 25 μL of 4 M HCl, which served as the initiator for the polymerization of PVA, was added to the vial. After approximately 2 min of sonication (to remove any air bubbles) the mixture was stirred vigorously for another 2 min. Polymerization to form the membrane was completed during this time. Once the polymerization reaction to form the membrane was completed, the entire contents of the vial were pipetted onto a glass slide for casting. After pipetting the contents onto a glass slide with a Teflon frame, a second glass slide was placed on top of it and clamped on all edges using portfolio clips to form the mold for membrane preparation. A suitable level of hardness was achieved by curing for one hour. The clips were then removed, and the cast was soaked in Petri-dish containing deionized (DI) water for one day. Once the desired level of hardness for the membrane was obtained, the membrane was removed by prying at the edges of the glass slides using a razor blade. Each membrane was checked for uniformity and irregular segments by measuring turbidity (700 nm) at every 5.0 mm [17]. The relative standard deviation between measurements taken across the membrane was less than 1.0%. Different segments from the same membrane generally gave the same results. Each membrane was stored in a 10 mL sample vial containing DI water, which was placed in a refrigerator. Further details about the preparation of the PVA membranes and the measurements used to assess membrane uniformity can be found elsewhere [17,27].

### 3.4. Turbidity Measurements

A Cary 6000i spectrometer (Varian Inc., North America) was used to study the swelling of the hydrogel membranes prepared from PVA and pH sensitive polyNIPA particles. Although absorbance spectra over the wavelength range 350–700 nm were collected for each membrane, turbidity profiles were constructed from the absorbance data collected at 700 nm. Measuring turbidity at longer wavelengths ensured that changes in the refractive index of the membrane is the dominant optical effect, as the intensity of the light scattered by the polymer particles decreases with the increasing wavelength of the incident light.

Each membrane was mounted onto a custom-built sample holder [17,27] constructed from black plastic to ensure that it would not pass stray light and would be chemically inert to the buffer solutions in contact with the membrane. A camera image of the “home-built” membrane holder can be found in Reference 17. To mount a polymer test membrane segment onto a sample holder, a membrane segment was placed in a Petri dish containing deionized water. The segment was then floated onto a microscope slide and smoothed to remove any wrinkles. The membrane was then attached to the inset piece of the sample holder by placing the piece on top of the membrane. This allows the membrane to completely cover the opening of the inset piece and to adhere to its plastic surface. The inset piece, which contains the membrane, was snapped onto the base unit of the sample holder. The complete sample holder, which includes both the base unit and inset piece, was placed into a conventional 1 cm path length cell sample cuvette. The remaining space in the cuvette was filled with deionized water or buffer solution. The cuvette was fitted to a custom-built flow cell to allow for the presentation of fresh buffer solution to the hydrogel membrane. The flow was regulated using a peristaltic pump (Anko Products, Bradenton, FL, USA) at a rate of 1 mL per minute.

### 3.5. Buffer Solutions

Buffers in the pH range 3.0–3.8 were prepared using chloroacetic acid/sodium chloroacetate. For the pH range 3.9–5.4, acetic acid and sodium acetate were used, MES was used to prepare buffer solutions in the pH range of 5.5–7.3, and MOPS was used for pH 7.4 to 8. In addition, the ionic strength of the buffers was adjusted by adding known amounts of NaCl to 0.05 M buffer solutions. The recipe for each pH buffer was calculated using an online buffer calculator [28].

## 4. Conclusions

Crosslinked polymers of NIPA copolymerized with an alkyl acrylic acid swell and shrink in response to changes in pH when placed in aqueous media. NIPA was selected as the major comonomer because copolymers of NIPA sensitized to pH using the appropriate functional comonomer undergo significant swelling and shrinking as a function of the pH of the solution in contact with the polymer. Furthermore, swelling is accompanied by a large change in the refractive index of the polyNIPA particles from a transparent state, where the refractive index of the particles and the solvent are equal, to a milky state resulting from the large amount of light scattered when the polymer is in its shrunken state.

Four pH-sensitive co-monomers were investigated in this study: acrylic acid, methacrylic acid, ethacrylic acid, and propacrylic acid. It is evident from the data presented that several of the polyNIPA particles investigated are good candidates for optical pH sensing in the lower portion of the physiological pH range. However, the pH response of the polyNIPA particles investigated showed a notable dependence on temperature and the ionic strength of the medium. Since acrylamide gels are complicated systems, decoupling their pH response is challenging. Fortunately, many potential application areas (including gastric pH sensing, in vivo pH sensing of blood and monitoring pH in fermentation baths and bioreactors over extended periods of time) occur in environments where the temperature and ionic strength of the sample exhibit only small variations. For these applications, calibrating the pH optrode at a given temperature and ionic strength which are in agreement with the physiological value of interest is feasible.

## Figures and Tables

**Table 1:** Formulation of Four Copolymers of *N*-isopropylacrylamide.

Component	M-78 Acrylic Acid (Millimoles)	M-70 Methacrylic Acid (Millimoles)	M-83 Ethacrylic Acid (Millimoles)	M-75 Propacrylic Acid (Millimoles)
*N*-isopropyl acrylamide	14.6	15	15	17
pH sensitive co-monomer	2	2	2	2
*N-tert*-butyl acrylamide	2	2	2	0
*N*,*N*-methylene bisacrylamide	1.4	1	1	1

**Table 2:** Apparent pK_a_ Values of the *N*-isopropylacrylamide Copolymers at 23 °C and 37 °C.

Apparent pK_a_	Acrylic Acid ^1^ M-78	Methacrylic Acid ^2^ M-70	Ethacrylic Acid ^3^ M-83	Propacrylic Acid ^4^ M-75
23 °C	3.9	4.3	5.3	5.5
37 °C	4.3	5.1	5.9	6.1

^1^ pK_a_ of acrylic acid at 25 °C, as determined by titration with NaOH standardized by KHP is 4.25. ^2^ pK_a_ of methacrylic acid at 25 °C, as determined by titration with NaOH standardized by KHP is 4.65. ^3^ pK_a_ of ethacrylic acid at 25 °C, as determined by titration with NaOH standardized by KHP is 4.65. ^4^ pK_a_ of propacrylic acid at 25 °C, as determined by titration with NaOH standardized by KHP is 4.84.

**Table 3:** ΔH and ΔS from pH-Induced Swelling of Four *N*-isopropylacrylamide Copolymers.

Enthalpy & Entropy	^2^ Acrylic Acid M-78	^2^ Methacrylic Acid M-70	^2^ Ethacrylic Acid M-83	^2^ Propacrylic Acid M-75
^1^ ΔH (J/mole)	−73,000 ± 8580	−88,700 ± 8230	−101,000 ± 2200	−90,400 ± 9500
^1^ ΔS (J/mole-K^0^)	−320 ± 28	−382 ± 27	−4440 ± 7.2	−410 ± 32

^1^ Uncertainties determined from the least squares fitting of the data. ^2^ see Table 1 for formulations.

**Table 4:** Formulations of *N*-isopropylacrylamide Copolymers of Ethyacrylic Acid.

Polymer	*N*-isopropyl-acrylamide (Millimoles)	Ethacrylic Acid (Millimoles)	*N-tert*-butyl Acrylamide (Millimoles)	*N*,*N*-methylene Bisacrylamide (Millimoles)
M-64	16.6	2	0	1.4
M-71	16.8	2	0	1.2
M-72	17	2	0	1
M-80	17	2	0	1
M-83	15	2	2	1

**Table 5:** pK_a_ Values of *N*-isopropylacrylamide Copolymers of Ethyacrylic Acid.

Apparent pK_a_	^1^ M-64	^1^ M-71	^1^ M-72	^1^ M-80	^1^ M-83
23 °C	4.9	4.7	4.7	4.9	5.3
37 °C	5.5	5.3	5.3	5.5	5.9

^1^ See Table 4 for the formulation.

**Table 6:** ΔH and ΔS of pH-Induced Swelling of *N*-isopropylacrylamide Copolymers of Ethacrylic Acid.

Enthalpy and Entropy	^1^ M-64	^1^ M-71	^1^ M-72	^1^ M-80	^1^ M-83
^2^ ΔH (J/Mole)	−81,700 ± 11,600	−82,000 ± 6200	−81,000 ± 10,000	−100,300 ± 3400	−101,000 ± 2200
^2^ ΔS (J/Mole-K^0^)	−370 ± 38	−370 ± 20	−360 ± 33	−430 ± 11	−440 ± 7.2

^1^ See Table 4 for the formulation. ^2^ Uncertainties determined from the least squares fitting of the data.

**Table 7:** Formulations of *N*-isopropylacrylamide Copolymers of Propacrylic Acid.

Polymer	*N*-isopropyl Acrylamide (Millimoles)	Propacrylic Acid (Millimoles)	*N-tert*-butyl Acrylamide (Millimoles)	*N*,*N*-methylene Bisacrylamide (Millimoles)
M-60A	16	2	0	2
M-62	16.6	2	0	1.4
M-74	16.8	2	0	1.2
M-75	17	2	0	1
M-101	18	1	0	1

**Table 8:** pK_a_ Values of *N*-isopropylacrylamide Copolymers of Propacrylic Acid.

Apparent pK_a_	^1^ M-60A	^1^ M-62	^1^ M-74	^1^ M-75	^1^ M-101
23 °C	5.9	5.7	5.7	5.5	5.3
37 °C	6.5	6.3	6.3	6.1	6.1

^1^ see Table 7 for the formulation.

**Table 9:** ΔH and ΔS of pH-Induced Swelling of *N*-isopropylacrylamide Copolymers of Propacrylic Acid.

Enthalpy and Entropy	^1^ M-60A	^1^ M-62	^1^ M-74	^1^ M-75	^1^ M-101
^2^ ΔH (J/mole)	−74000 ± 11300	−73300 ± 8400	−74900 ± 8000	−90400 ± 9500	−95400 ± 8100
^2^ ΔS (J/mole-K^0^)	−361 ± 37	−360 ± 27	−370 ± 26	−411 ± 32	−420 ± 27

^1^ see Table 7 for the formulation. ^2^ Uncertainties determined from the least squares fitting of the data.
